# Two-Dimensional Mechanics of Atomically Thin Solids on Water

**DOI:** 10.1021/acs.nanolett.2c02499

**Published:** 2022-09-01

**Authors:** Jaehyung Yu, Ce Liang, Myungjae Lee, Soumik Das, Andrew Ye, Fauzia Mujid, Preeti K. Poddar, Baorui Cheng, Nicholas L. Abbott, Jiwoong Park

**Affiliations:** † Department of Chemistry, University of Chicago, Chicago, Illinois 60637, United States; ‡ Pritzker School of Molecular Engineering, University of Chicago, Chicago, Illinois 60637, United States; § James Franck Institute, University of Chicago, Chicago, Illinois 60637, United States; ∥ Department of Chemical and Biomolecular Engineering, Cornell University, Ithaca, New York 14853, United States

**Keywords:** Micro/nano mechanics, air−water interface, 2D materials, MoS_2_

## Abstract

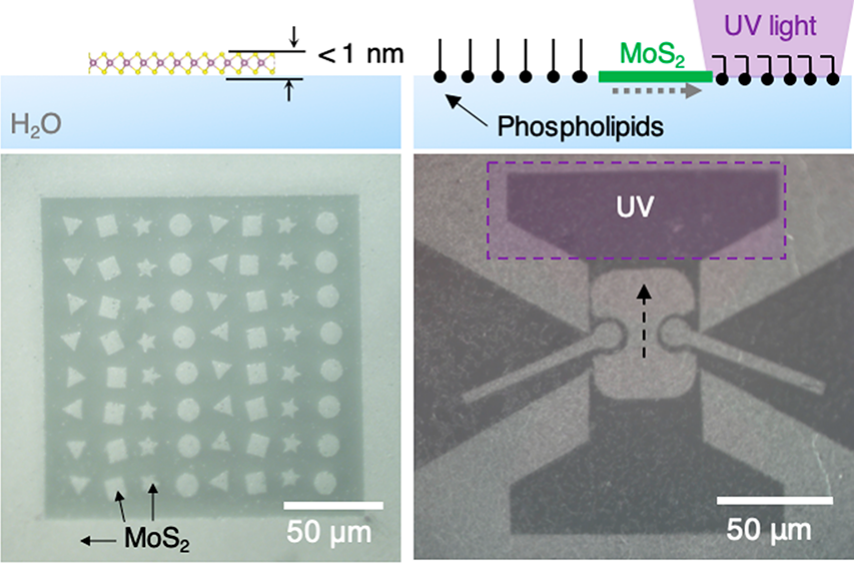

Movement of a three-dimensional solid at an air–water interface is strongly influenced by the extrinsic interactions between the solid and the water. The finite thickness and volume of a moving solid causes capillary interactions and water-induced drag. In this Letter, we report the fabrication and dynamical imaging of freely floating MoS_2_ solids on water, which minimizes such extrinsic effects. For this, we delaminate a synthesized wafer-scale monolayer MoS_2_ onto a water surface, which shows negligible height difference across water and MoS_2._ Subsequently patterning by a laser generates arbitrarily shaped MoS_2_ with negligible in-plane strain. We introduce photoswitchable surfactants to exert a lateral force to floating MoS_2_ with a spatiotemporal control. Using this platform, we demonstrate a variety of two-dimensional mechanical systems that show reversible shape changes. Our experiment provides a versatile approach for designing and controlling a large array of atomically thin solids on water for intrinsically two-dimensional dynamics and mechanics.

A solid object freely floating on water is described by the dynamics of a two-dimensional (2D) system. Its motion confined at the air–water interface is largely described with three degrees-of-freedom–two lateral center-of-mass coordinates and one rotation angle–instead of six for 3D systems. However, introducing a solid with a finite thickness or a nonflat shape onto a water surface (Figure 1a, top) displaces water and bends the water surface due to the solid’s mass, buoyancy, and the surface tension.^1^ This strongly affects the dynamics of a floating solid and creates additional forces between them. Specifically, moving a solid on water is harder as it induces a flow of water around it, and the curved water surface around each solid induces a capillary force that attracts neighboring solids to form clusters spontaneously.^2−4^ Thus, an accurate description of multiple solids on water requires a full understanding and control of the interplay between their masses, shapes, and the interfacial interactions.^5,6^ This also suggests that this intricate picture becomes significantly simpler in the limit where the floating solid is flat and atomically thin. In this limit, the amount of displaced water and surface curvature become negligible, which makes this an ideal 2D mechanical system, as described schematically in Figure 1a, bottom.

**Figure 1:**
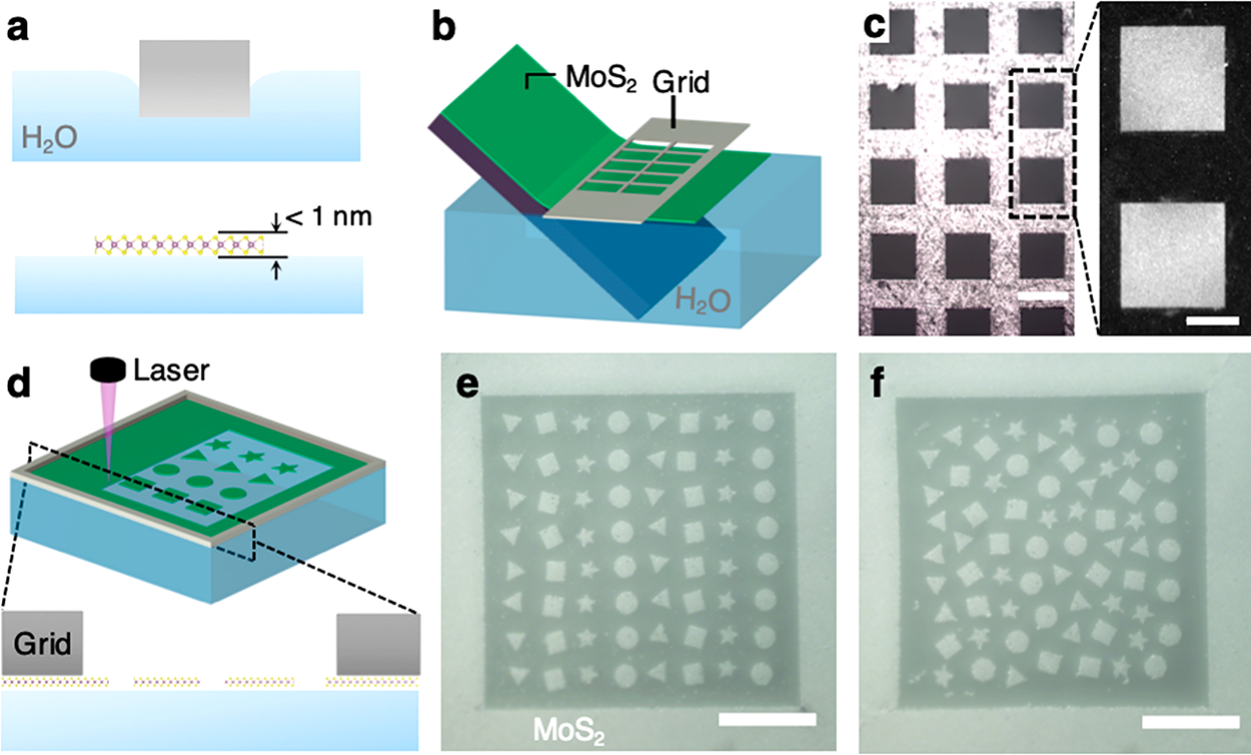
Fabrication of monolayer MoS_2_ solids on water surface. (a) Schematic of water surface with 3D solid (top) and 2D solid (bottom) object. (b) Schematic of floating MoS_2_ membrane array fabrication on water surface. (c) Optical reflection (left) and photoluminescence emission (right, λ_em_ = 680 nm) images of floating 2D membrane array tethered to metal grid. Scale bar = (left) 200 μm, (right) 100 μm. (d) Schematic of laser patterning on floating MoS_2_ membrane. (e,f) Optical reflection image of free-floating MoS_2_ solids right after (e), and 20 min after (f) laser patterning. Scale bar = 50 μm.

Creating and studying such 2D mechanical systems on water composed of atomically thin solids would require three key capabilities. First, one needs to generate atomically thin, flat solid films compatible with water. Second, they need to be patterned into arbitrary lateral shapes with minimal effects to their original locations and properties. Third, there needs to be a mechanism for exerting lateral forces to these solids with spatial and temporal control. Monolayers of 2D layered materials, such as graphene and monolayer transition metal dichalcogenides (TMDs), are ideal candidates for this purpose, because of their atomic-scale thinness, their flat topography, and their stability in and on water.^7−11^ They are also mechanically strong with high in-plane mechanical moduli and tear resistance, allowing them to be freely suspended in vacuum and used as solid membranes on water.^12−15^ Although there exist studies of 2D materials on water,^16,17^ none of these previous reports demonstrate all the necessary capabilities for generation, patterning, and control of 2D solids on water.

Here, we present 2D mechanical systems based on atomically thin and flat TMD monolayers, that are systematically generated, patterned, and controlled over a large scale while on water. We first synthesize wafer-scale monolayers of MoS_2_, a representative TMD, that are continuous and uniform on a flat SiO_2_-Si substrate. MoS_2_ is gently delaminated onto a water surface and anchored by a rigid frame to generate an array of flat and stable MoS_2_ on water without cracks and wrinkles. Then a scanning laser is used to fabricate 2D solids with intended shapes in a submicrometer resolution. Finally, photoswitchable surfactant is introduced to the exposed water surface not covered by MoS_2_ which enables us to generate a local surface energy difference and lateral force by shining spatially controlled light. We use these experimental capabilities to produce 2D mechanical systems composed of microscopic MoS_2_ solids on water and demonstrate reversible linear translocation, rotation, and lateral shape changes, as described below in detail.

Figure 1 explains our process for generating an array of patterned MoS_2_ solids on water with representative results. Monolayer MoS_2_ is first synthesized by metal–organic chemical vapor deposition (MOCVD) on a 2 in. SiO_2_–Si substrate with uniform and continuous coverage.^18^ This as-grown monolayer MoS_2_ on the growth substrate is placed, tilted at an angle ∼45°, in a container with a metal grid frame (see the schematic, Figure 1b), where deionized water is slowly introduced. As the water level rises above the lower end of the substrate, MoS_2_ becomes gradually delaminated from the substrate onto the water surface. The delamination process is continued until the MoS_2_ contacts the metal grid frame from underneath, which anchors the membrane for further patterning, imaging, and control (see Methods (Supporting Information) and Figure S1 for more details). Figure 1c shows optical reflection (left) and photoluminescence (PL, right) images of an example MoS_2_ array on water, where MoS_2_ appears darker (brighter) than the frame in the reflection (PL) image. They confirm that our process successfully produces a continuous MoS_2_ monolayer membrane supported by water, tethered inside an array of large, 0.2 mm by 0.2 mm, windows. The homogeneous PL intensity in Figure 1c, which is measured near the MoS_2_ bandgap emission wavelength, also confirms the uniformity and continuity of the floating MoS_2_ membranes without mechanical deformations (e.g., wrinkles) or cracks.

A scanning, computer-controlled laser beam (532 nm) with a diffraction-limited spot (<1 μm) is then used to pattern the MoS_2_ membrane on water (see Figure 1d).^19^ This enables us to fabricate an array of MoS_2_ solids with arbitrary shapes and spacings surrounded by a boundary also defined using MoS_2_, without using conventional lithography process. Thus, our system ensures that every component, including the patterned solids, water, and the boundary, remains flat across the entire surface. Figure 1e shows an optical image of an example 8 by 8 array of MoS_2_ solids (bright) on water (dark) right after patterning (see also Supporting Information Video 1). They have varying shapes, including triangles, circles, squares, and stars, all patterned according to the intended microscopic design. Over time, these patterned MoS2 solids continuously move to different locations (see Figure 1f, taken after 20 min, and Supporting Information Video 2). We note that there is no aggregation or lateral sticking among neighboring MoS_2_ solids, unlike the case of thicker solids or lithographically patterned 2D materials on water.^16^

The above results confirm that our approach successfully produces an array of atomically thin, freely moving MoS_2_ solids with predesigned microscopic shapes on water. The entire surface is expected to remain flat by design, as they are composed of liquid water and monolayer MoS_2_, which is also grown flat. This is indeed what we observe based on the height images taken using a confocal laser scanning microscope (see Figure 2 and Methods in Supporting Information). Figure 2a shows a zoomed-out height map of an example window of patterned MoS_2_ (design shown in Figure 2b, inset) on water (middle lower region) surrounded by the tall metal frame (thickness ∼10 μm). Figure 2b shows a zoomed-in image of only the patterned MoS_2_ and water surface with a much smaller vertical scale of 100 nm. Even though the MoS_2_ and water surface are clearly visible in the reflection image (inset),^20^ they are nearly indistinguishable in their corresponding confocal height images. The cross-sectional height profile in Figure 2c further confirms that the height difference across multiple regions of water and MoS_2_ solids remains within the resolution of the instrument (12 nm; marked by the gray area). This also suggests that there will be negligible out-of-plane bending to water surface near the edges of MoS_2_ solids and no capillary forces.

**Figure 2:**
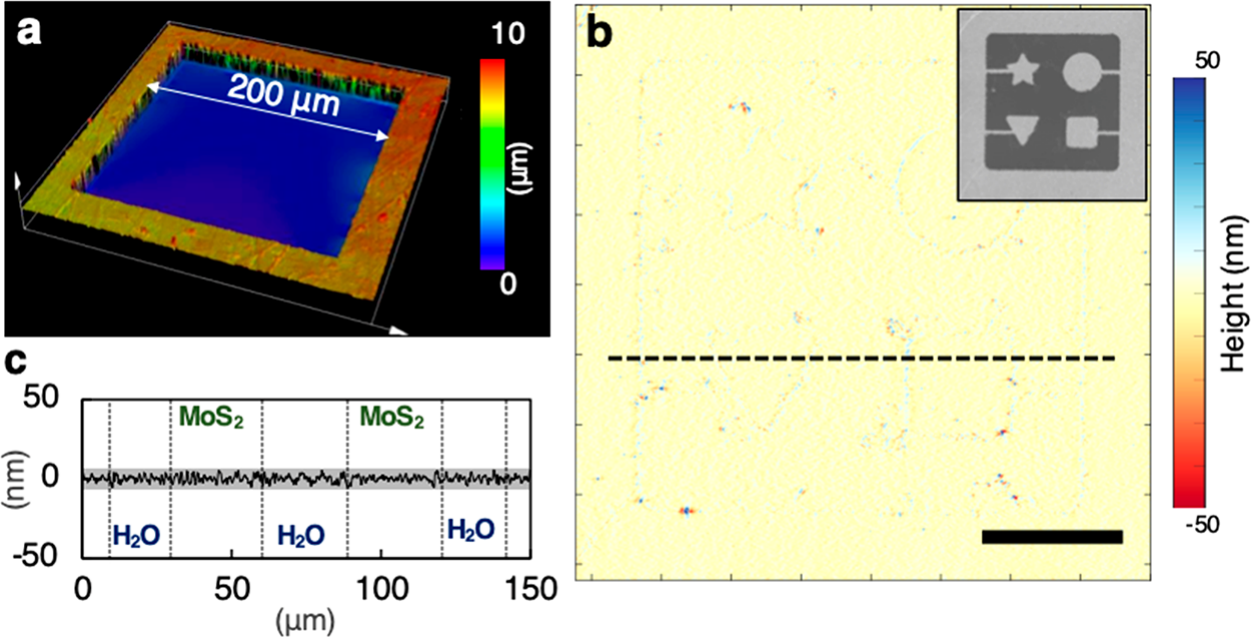
Surface topography of patterned MoS_2_ on the water surface measured by confocal laser scanning microscope. (a) Three-dimensional height image measured by confocal laser scanning microscope including metal grid. (b) Magnified height profile of patterned MoS_2_ on the water surface. Inset shows the reflection image of the corresponding pattern. Scale bar = 40 μm. (c) Cross-section height profile (bottom) corresponds to the dashed line with the resolution (12 nm) shown as gray area.

Another advantage of our approach based on the gentle delamination and tethering by the frame is that it produces MoS_2_ membranes with minimal in-plane strain. This is necessary for accurate and reliable pattern transfer, because a MoS_2_ membrane, if strained, will expand or shrink after patterning. Figure 3a (b) compares the Raman (PL) spectra measured from a MoS_2_ membrane as grown on SiO_2_ (dotted line) with the spectra of the same MoS_2_ on water (solid line). After delamination, both the Raman (ΔE_2g_ = +2.01 cm^–1^ and ΔA_1g_ = +2.51 cm^–1^) and PL (+20 meV) peaks shift relative to those from as-grown MoS_2_, which suggest that approximately 0.2% tensile strain is released by delamination. This amount is comparable to the tensile strain (0.24%) present in the as-grown MoS_2_, estimated based on the thermal expansion coefficient mismatch (SiO_2_ vs MoS_2_) during the cooling-down from the high MOCVD synthesis temperature (525 °C).^21−23^ Our data also show that the strain distribution of the floating MoS_2_ is more homogeneous, as both Raman and PL peaks are narrower on water (insets, Figures 3a,b). This suggest that the MoS_2_ membrane on a SiO_2_–Si substrate is under a tensile strain, which is released by delaminating MoS_2_ to the water surface.

**Figure 3:**
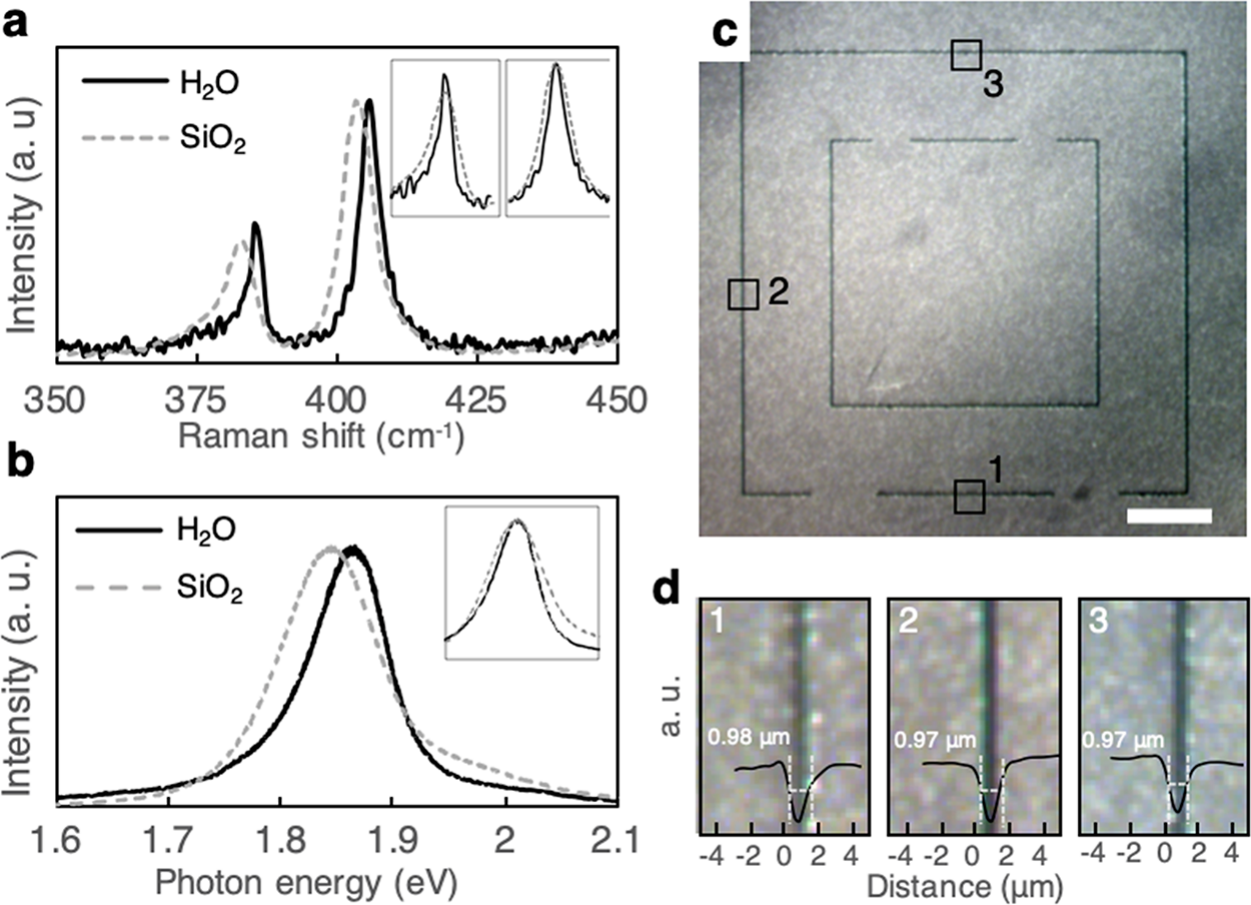
Measuring residual in-plane strain of floating monolayer MoS_2_ on water. (a,b) Raman (a) and PL (b) spectrum of the monolayer MoS_2_ on SiO_2_ (dotted line) and water (solid line). Insets compare the width of each peak on both substrates. (c) Optical image of floating MoS_2_ with line cuts. Scale bar = 25 μm. (d) Magnified optical images of line cuts marked in (c) and averaged profile of corresponding line cuts extracted from the image.

Figure 3c confirms that our MoS_2_ membrane on water does not change its size or shape after patterning. It shows an optical reflection image of a patterned MoS_2_ membrane on water, which contains nesting MoS_2_ squares each tethered to a larger square on one side. They are patterned and separated by a single line scan using the focused laser beam with a diffraction limited spot. The average widths of the four straight lines (top, bottom, left and right) are almost identical (0.98 ± 0.01 μm, see Figure 3d and Figure S2), which shows that the MoS_2_ membrane is patterned almost perfectly square without expansion, compression, or shear. This suggests that our delamination and patterning process can generate micrometer sized MoS_2_ solids with accurate sizes and shapes (see Figure S3). This is not the case if we reverse the order by patterning first and then delaminating the MoS_2_. In such a case, wrinkles and cracks are formed around the patterned lines, and the widths of lines become irregular (see Figure S4).

Actively controlling the two-dimensional motion of our fabricated MoS_2_ solids beyond their free motion requires generating forces that can act on them laterally (parallel to the air–water interface) but not vertically. For this, we utilize functionalized lipids, which can be distributed uniformly on the water surface not covered by the MoS_2_ (See Figure 4a and Methods in Supporting Information).^24−28^Figure 4b shows optical reflection (top) and PL (bottom) images of patterned MoS_2_ solids after adsorbing fluorescent lipids onto the water surface from the subphase. The lipid PL (green) appears in regions of the surface not occupied by MoS_2_, which indicates that the lipid molecules and floating MoS_2_ solids coexist on the air–water interface, even in the enclosed boundaries.

**Figure 4:**
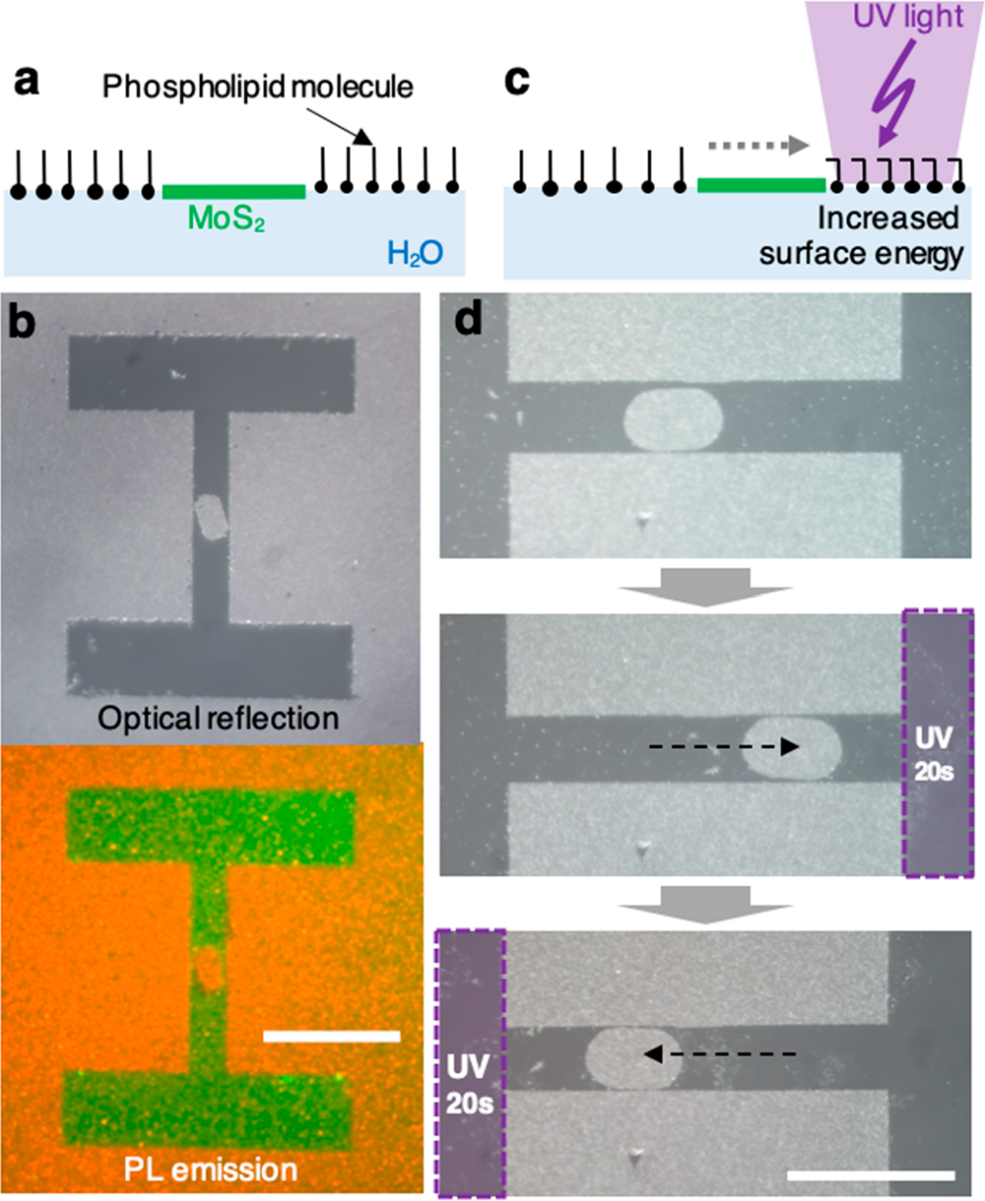
Distributing functionalized lipids to exert lateral forces on floating MoS_2_. (a) Schematic of the phospholipid distribution with floating MoS_2_. (b) Optical (top) and photoluminescence (bottom) emission image of MoS_2_ (red, λ = 680 nm) and fluorescent surfactant (green, λ = 530 nm). (c) Schematic of the photolipid-mediated actuation by locally illuminating UV light. (d) Optical reflection images of back-and-forth translocation of MoS_2_ solids. The purple area shows the location of UV illumination. All scale bar = 50 μm.

A photoswitchable lipid is then used to change the surface energies of different region of the water surface by light illumination. Such surface energy difference generates a lateral force, also known as Marangoni force (See schematic, Figure 4c), that acts on the MoS_2_.^29,30^ In our experiment, we illuminate UV (365 nm) and blue (430 nm) light, which induces reversible cis–trans isomerization of azo-benzene in the tail of the lipid molecule.^31−33^ Shining UV light on a particular region increases its surface energy, and if only one side of a MoS_2_ solid is illuminated, it will be effectively pushed toward the illuminated region. This is demonstrated in Figure 4d. A MoS_2_ solid is first placed in the middle of a channel (top image); it moves toward right when the right side (marked as purple squares) is illuminated by UV light (∼10 W/m^2^ for 20 s; middle image); then it moves toward left with the UV light on the left side (bottom image). The MoS_2_ particle starts to move immediately when the UV illumination is initiated and the displacement of the particle linearly increases with the UV exposure time, suggesting a constant speed (∼0.8 μm/s) upon actuation. This speed changes depending on the quantity of photoswitchable phospholipids in the system and the UV illumination intensity (see Figure S5). Between the middle and bottom images, we introduce blue light for 1 min to equilibrate the surface energy. The magnitude of force that can be generated using this method can be estimated from past studies, which have reported that azobenzene-based phospholipids can change the surface energy of water upon UV illumination by 15 mJ/m^2^. Based on this, we estimate a lateral force of ∼0.3 μN on the MoS_2_ solid in our experiments.

The data in Figure 4 fully demonstrates reversible linear translocation of aMoS_2_ solid, driven by a lateral force generated by the illumination of UV and blue lights. Using the same principle, we can design complex structures that display a variety of programmed motions and shape changes, as shown in Figure 5. Figure 5a presents a 2D mechanical system based on three MoS_2_ parts that simultaneously show translocation and rotation. It includes the main piston, two sides arms, and the hinges that confine the arms without attaching them to the piston. Illuminating UV light on the bottom side pushes the piston that direction (see the middle panel), while the left (right) arm rotates clockwise (counterclockwise). A cyclic illumination of UV and blue light on the structure’s top and bottom region drives the piston up or down (linear translocation), while the side arms swing clockwise or counterclockwise (rotation).

**Figure 5:**
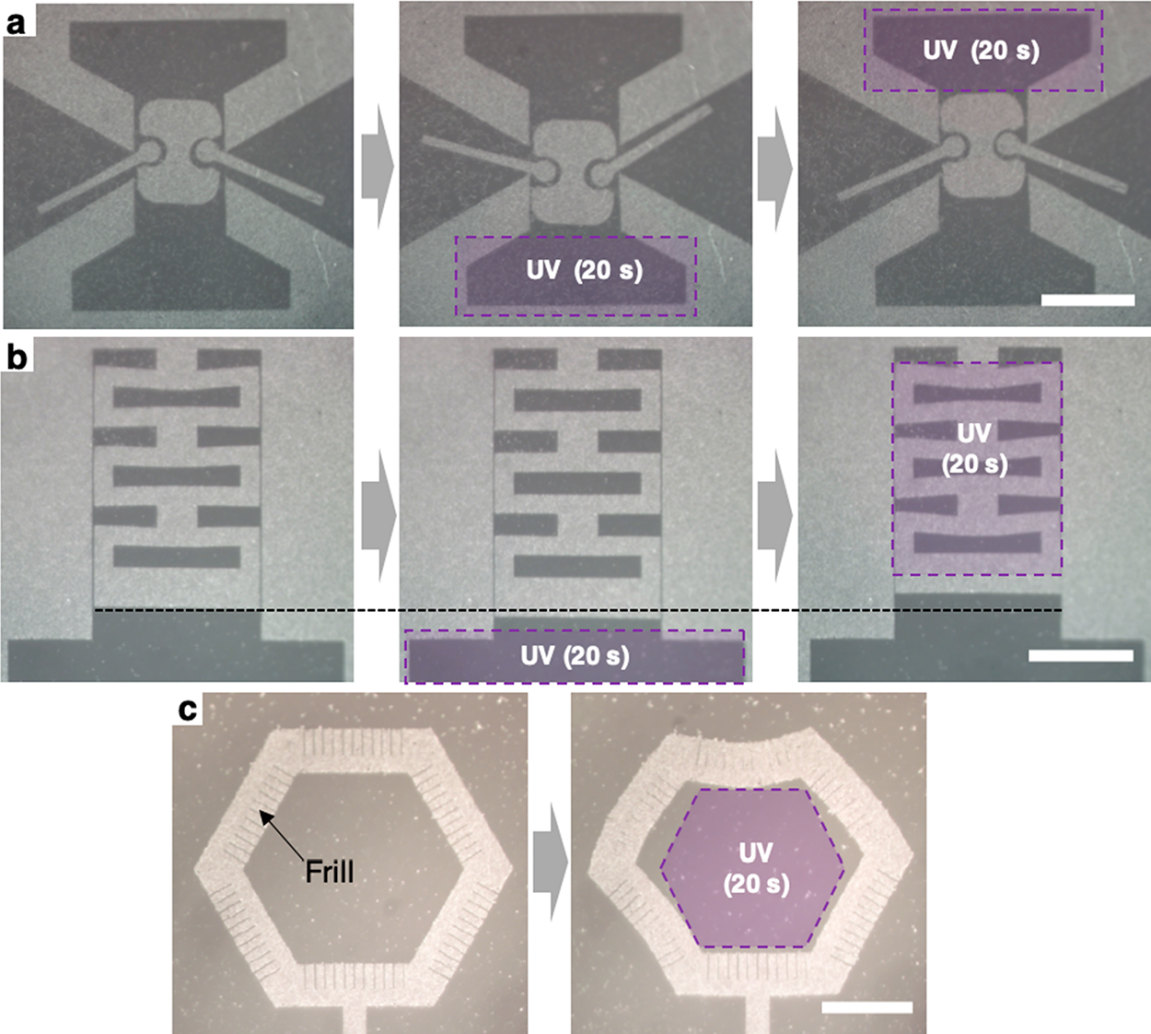
Optical reflection images of cyclic translocation and shape changes of MoS_2_ solids. (a) Cyclic translocation of multiple bodies. (b) From the neutral position(left), elongation (middle) and compression (right) of 2D kirigami spring. The dotted line marked for comparing length. (c) Compressive deformation of hexagonal ring with frills. The purple area shows the location of UV illumination for 20 s. Between the sequence of each image, 1 min of blue light is introduced to the image area. All scale bar = 50 μm.

Figure 5b demonstrates a 2D spring that reversibly stretches and compress. Compared to the neutral length (left), the 2D spring is elongated as much as 5% when we illuminate UV light at the bottom (middle). Subsequently, ∼5% compression is induced by shining UV on the spring region after resetting the surface energies using blue light (right). Such strain variation is reversibly seen after multiple cycles, and the spring comes back to the neutral position after few minutes from a deformed state in the absence of light illumination. This suggests that the nature of the deformation is elastic, not plastic, most likely accompanied the out-of-plane bending of some parts of the MoS_2_.^11^ We also note that such in-plane shape change becomes more difficult as the width of the MoS_2_ strip grows larger, as can be seen from the bottom MoS_2_ strip that remains straight throughout Figure 5b.

This lateral mechanical rigidity of a wide MoS_2_ strip can be reduced by introducing multiple line cuts, referred to here as frills. Figure 5c shows a hexagonal MoS_2_ ring with each MoS_2_ bar having frills on one side (alternating between inside and outside). Its shape is then deformed by UV illumination (right image). We observe that the in-plane bending deformation only occurs in the direction of closing the frills. Interestingly, their deformed shapes remain even after resetting the surface energies by shining blue light, and the high resolution optical images of frills (Figure S6) show additional lines with sharp contrast. Based on these observations, we speculate that the frills offer nucleation points for local out-of-plane buckling of the MoS_2_ on water. The high contrast lines are similar to the features previously associated with out-of-plane buckling instabilities formed in compressed monolayer MoS_2_,^34^ which accommodate a global strain with localized out-of-plane bending, reducing the total elastic energy.^35^ Moreover, the formation such buckling instabilities is not reversible, consistent with what we observe in Figure 5c. The existence of frills also softens the floating MoS_2_ under 2D bending deformation. The in-plane bending stiffness in Figure 5c is estimated to be 1.6 × 10^–15^ Nm^2^, which is approximately 1 order of magnitude smaller than the theoretical estimate (1.43 × 10^–14^ Nm^2^) based on its Young’s modulus of MoS_2_^36^ (see Supporting Figure S7 in detail). In contrast, the bending stiffness obtained from Figure 5b (1.2 × 10^–14^ Nm^2^) is much closer to the theoretical estimate.

In summary, we report a scalable and actuatable mechanical system based on atomically thin solids floating on the surface of water. This can provide an ideal platform for realizing, investigating, and controlling the 2D mechanics of solids in the regime that was previously inaccessible. This includes establishing the principle for inducing controllable three-dimensional, out-of-plane buckling of 2D solids, which could be useful for designing microscale mechanical applications, such as floating micromechanical logic gates or surface microswimmers.^37,38^ Another example is to quantify and control the force and the interactions (e.g., electrical, magnetic) among 2D solids. In fact, our data (see Supporting Information Video V2) suggest that the motions of floating 2D solids are sensitive to the presence of any forces between solids. Finally, it would allow us to measure the friction and drag at the 2D solid–water surface. These studies, when combined, will enable us to understand and manipulate floating 2D solids using various external stimuli, such as electrostatic charging, thermal gradients, catalytic propulsion, and surface acoustic waves.^39−44^

## Supporting Information Available

Methods for detailed experimental information, and additional figures (PDF)
Video for *in situ* laser patterning of 2D solids on water (MP4)
Video for free motion of 2D solids on water (MP4)
