# Supplementary material for: Two-Dimensional Mechanics of Atomically Thin Solids on Water
Source: Nano Lett. 2022 Sep 1;22(17):7180–6. doi: 10.1021/acs.nanolett.2c02499 (PMC9479134; doi:10.1021/acs.nanolett.2c02499)
Supplement: Supplementary file 1 — nl2c02499_si_001.pdf [file nl2c02499_si_001.pdf]

Supporting Information:

Two-dimensional mechanics with atomically thin solids on water

Jaehyung Yu[1], Ce Liang[2], Myungjae Lee[3], Soumik Das[4], Andrew Ye[2], Fauzia Mujid[1], Preeti K. Poddar[1], Baorui Cheng[1], Nicholas L. Abbott[4], Jiwoong Park[1,2,3,*]

[1] Department of Chemistry, University of Chicago, Chicago, IL 60637, USA

[2] Pritzker School of Molecular Engineering, University of Chicago, Chicago, IL 60637, USA

[3] James Franck Institute, University of Chicago, Chicago, IL 60637, USA

[4] Department of Chemical and Biomolecular Engineering, Cornell University, Ithaca, NY 14853, USA

[*] Corresponding author. Email: jwpark@uchicago.edu

1. Methods

Fabrication of floating 2D membrane platform. The homogeneous monolayer MoS_2 is synthesized on the 2-inch SiO_2-Si substrate with MOCVD. The as-grown MoS_2 on the substrate is placed tilted 45° on the water bath of 150 mm(W) x 60 mm (L) x 60 mm (H). To delaminate the MoS_2 monolayer onto the water surface as gently as possible, we introduce DI water into the bath to raise the water level at a constant rate less than 1.5 mm/hr. As the water level rises, water intercalates between the MoS_2 and the SiO_2-Si substrate, which then begins to suspend the monolayer MoS_2 on the water (see supporting figure S1). The DI water is introduced until the water surface and MoS_2 membrane contact the grids(Nickel-Brass shadow mask, Photo-sciences). The size of grid is 4 cm (W) x 1 cm, with an array of 200 μm sized holes. After the suspended membrane touches the grid, the membrane with the grid is transferred to a small, portable water container(20 mm (W) x 25 mm (L) x 5 mm (H)) for further material characterizations and laser patterning.

In-situ direct laser patterning on the floating MoS_2 membrane. To achieve the diffraction-limit resolution patterning, we optically couple the laser scriber (Keyence MD-T1010W) to an upright optical microscope (Olympus BX-51). We apply the 4f-optics principle to reduce the laser beam spot down to the diffraction limit.

During the patterning, the MoS_2 is exposed to the high-power 532 nm laser with an average power density of 2.6 GW/m^2 with Q-switch frequency of 300 kHz. The scanning rate of laser is 50mm/s with the line spacing of 0.5μm to completely ablate the floating MoS_2 membrane.

Topography measurement with the confocal laser scanning microscope. We utilize an Olympus LEXT OLS 5000 to measure the surface topography of floating MoS_2 on the water surface. To measure the surface topography in high accuracy, we use specific 100x objective lens designed for the microscope (MPLAPON100xLEXT), which has numerical aperture of 0.95. The surface topography of both the water surface and the edge of grids are measured by detecting the

reflection intensity change of the confocal laser (405 nm) from the surface while shifting the z-axis focus position. The minimum resolution of the microscope is 12 nm.

Formation of phospholipid monolayers. We used fluorescent phospholipid (16:0-06:0 NBD PC, Avanti Polar Lipids) (in Fig. 3) or photo-switchable phospholipid (18:0 Azo PC, Avanti Polar Lipids) as the functionalized lipids (in Fig. 4,5). The lipids were mixed with cetyltrimethylammonium bromide (CTAB) in water to form mixed micelles of lipid and CTAB. The micelles increase the solubility of phospholipids for delivery to the exposed water surface.[1–3] We first dissolve the phospholipid in chloroform in a vial. The chloroform is evaporated and an aqueous solution of CTAB is then introduced in the vial. The surfactant/lipid solution is made by mixing the functionalized phospholipids and CTAB at a molar ratio of 1:20 to obtain a final lipid concentration of 63.7 μM in water. We introduced 50 μl of this solution into the water bath (~2.5 mL) with an exposed surface area of ~400 mm^2. The solution is then allowed to equilibrate for at least 24 hours to distribute the lipids uniformly on the water surface. In a separate experiment, we also exchanged the aqueous solution containing CTAB with an aqueous solution free of CTAB. This process ensures the formation of a densely packed monolayer of the lipid with a molecular area of approximately 20 $Å^2$ – 30 $Å^2$.

UV actuation of MoS_2. To induce the 2D pressure with the functionalized phospholipids, we used Mercury lamp with band path filters for UV light (365 ± 15 nm) and blue light (430 ± 10 nm) linked through the Olympus BX-51 optical path. The local illumination of the light is achieved by utilizing the field stop in the optical microscope. The surface pressure of the Azo PC decorated interface is dynamically regulated by the wavelength of the light used. Based on observations from past works, we estimate that when illuminated by UV light of wavelength 365 nm, the surface pressure decreases (surface energy increases) by approximately 15 mN/m, following isomerization from the *trans* to the *cis* state.[4]

2. Supporting figures

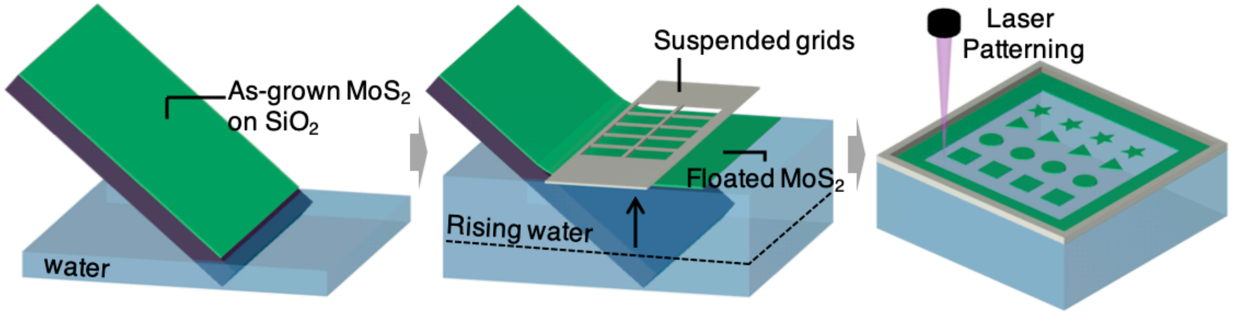

Supporting Figure S1. Schematic of experimental procedures for floating 2D solids fabrication process.

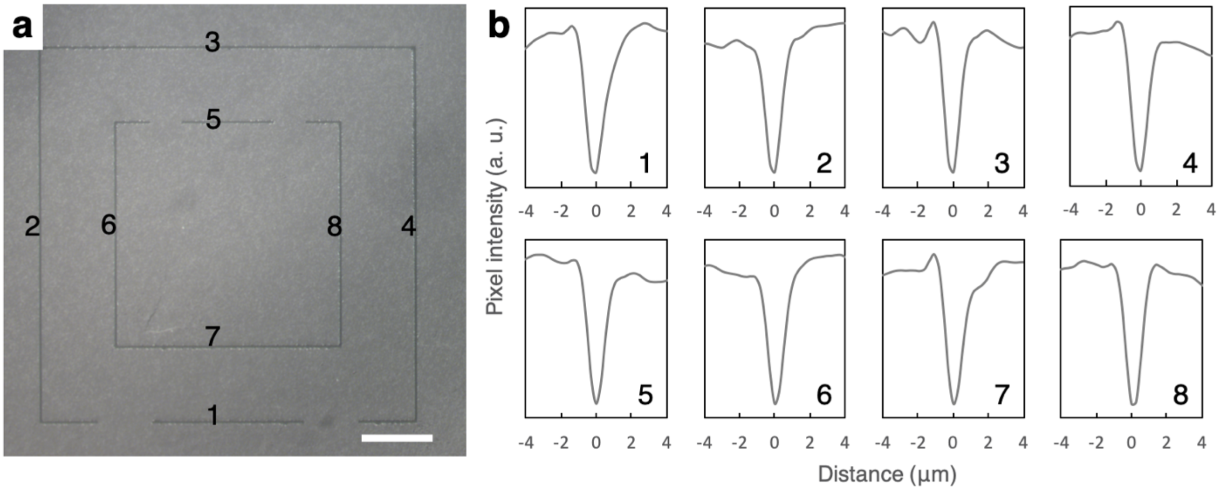

Supporting Figure S2. Measurement on the width of line cuts. **(a)** Optical image of floating MoS_2 with line cuts, and **(b)** corresponding line profiles. The line profile is extracted from the pixel intensity of the image, and width is measured as full-width half max (FWHM) of the peaks. Scale bar = 25 μm

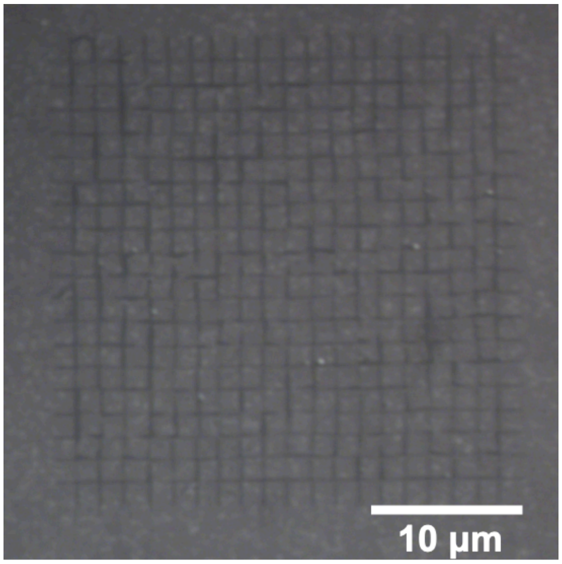

Supporting Figure S3. Diffraction limit patterning of floating MoS_2 membrane. The size of each square is < 2 µm.

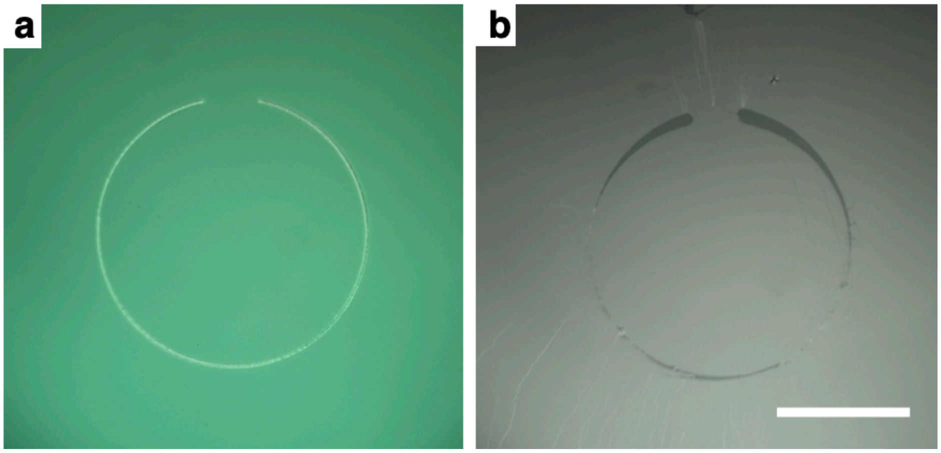

Supporting Figure S4. Floating monolayer MoS_2 after patterning line. **(a)** Optical image of circle line patterned to the MoS_2 on SiO2-Si substrate. **(b) O**ptical image of same circle line after floating on water surface. On water, the shape of circle lines is distorted. Furthermore, wrinkles and cracks are formed around the edge of circle line. Scale bar = 100 μm.

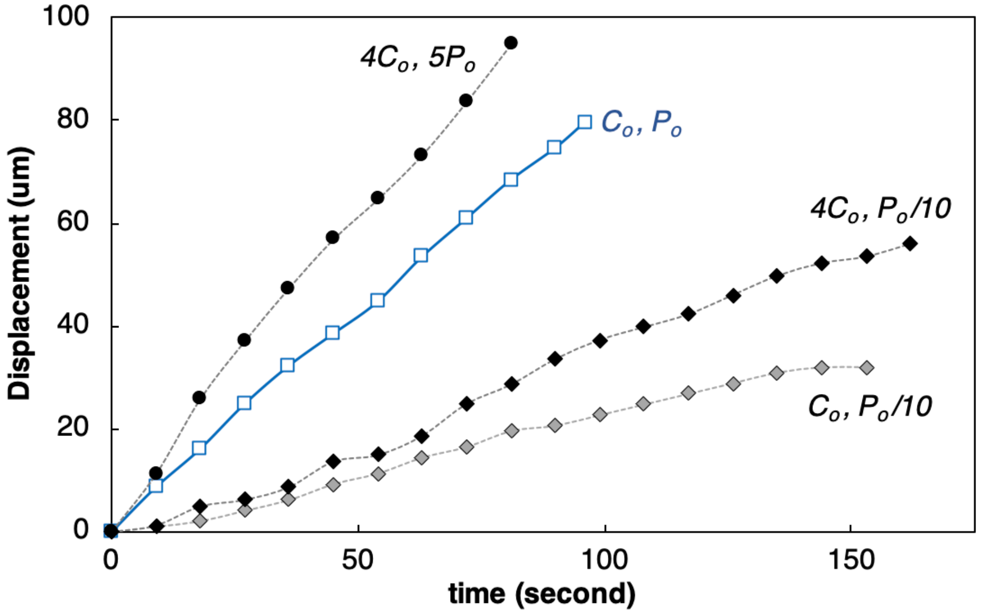

Supporting Figure S5. The displacement of MoS_2 solid as a function of UV exposure time under different experimental conditions of *C* (photlipid amount in the bath) and *P* (UV intensity). The Blue lines with square markers correspond to the original configuration shown in Fig. 4d; C_o = 50 μl and P_o = 10 W/m^2.

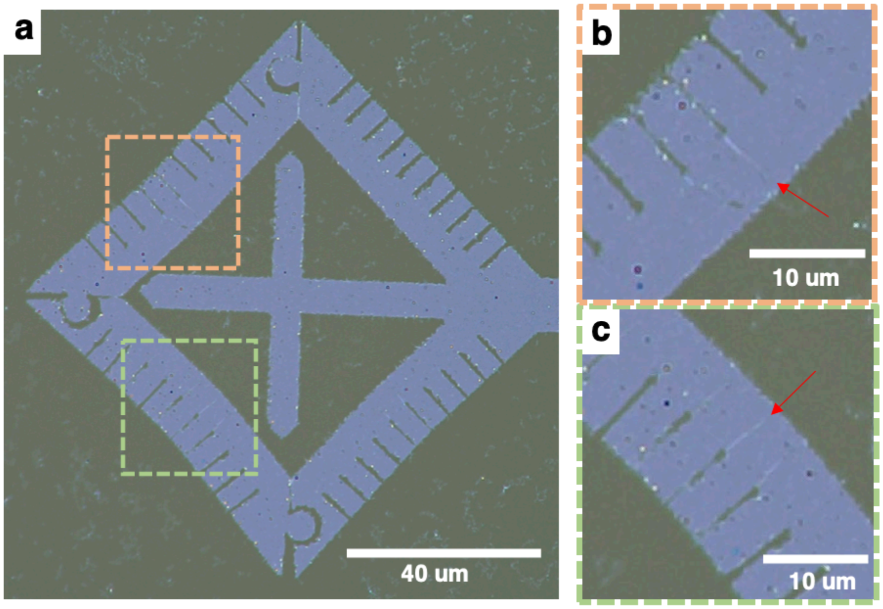

Supporting Figure S6. Formation of localized out-of-plane buckling on frill structure. **(a)** Optical reflection image of floating 2D micro-solid with frill structure on sides. UV illumination on the middle induces in-plane bending deformation. **(b, c)** Magnified optical image of frill structures marked in (a) while in-plane bending deformation. Red arrow shows the out-of-plane buckling instabilities due to the focused stress during in-plane bending happens.

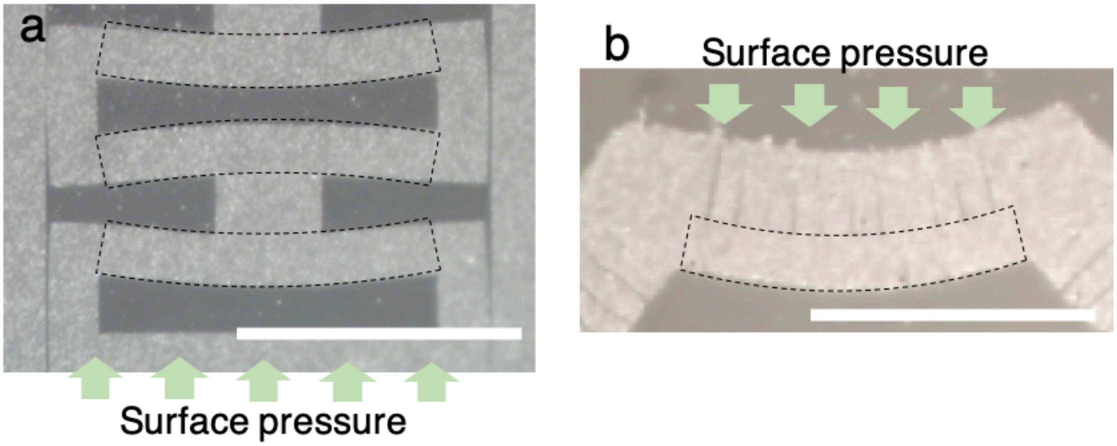

Supporting Figure S7. The modeling of in-plane bending in (a) Figure 5b and (b) Figure 5c. Dotted lines outline the MoS_2 structure is subjected under the in-plane bending deformation. The in-plane bending stiffness is estimated by the simple beam bending model with constant surface pressure (15 mN/m) applied to the beam. Scale bar = 50 μm.

Reference

1. Tiberg, F., Harwigsson, I. & Malmsten, M. Formation of model lipid bilayers at the silica-water interface by co-adsorption with non-ionic dodecyl maltoside surfactant. *Eur. Biophys. J.* **29**, 196–203 (2000).
2. Brake, J. M., Daschner, M. K., Luk, Y. Y. & Abbott, N. L. Biomolecular Interactions at Phospholipid-Decorated Surfaces of Liquid Crystals. *Science (80-.).* **302**, 2094–2097 (2003).
3. Almgren, M. Mixed micelles and other structures in the solubilization of bilayer lipid membranes by surfactants. *Biochim. Biophys. Acta - Biomembr.* **1508**, 146–163 (2000).
4. Backus, E. H. G., Kuiper, J. M., Engberts, J. B. F. N., Poolman, B. & Bonn, M. Reversible optical control of monolayers on water through photoswitchable lipids. *J. Phys. Chem. B* **115**, 2294–2302 (2011).
